# Enhancing Self-Efficacy Through Robotic Safety Support in Balance-Challenging Reach Tasks: Feasibility Study in Young Adults

**DOI:** 10.2196/81263

**Published:** 2025-09-30

**Authors:** Daiki Shimotori, Soshi Fujisawa, Masahiro Nishimura, Tatsuya Yoshimi, Kenji Kato

**Affiliations:** 1 Laboratory of Practical Technology in Community National Center for Geriatrics and Gerontology Obu Japan; 2 Laboratory of Clinical Evaluation with Robotics National Center for Geriatrics and Gerontology Obu Japan

**Keywords:** robotics, self-efficacy, fall-related self-efficacy, preventing falls, postural balance, assistive technology, human–robot interaction, functional reach test, psychological reassurance, rehabilitation

## Abstract

**Background:**

Falls and fear of falling adversely affect the quality of life and independence of older adults. Although various robotic systems have been developed for fall prevention, their psychological effects, particularly on self-efficacy, remain underexplored. A ceiling-mounted fall impact mitigation robot offers continuous protection with almost no limitations on the range of movement; however, its impact on users' psychological state and functional performance is unclear.

**Objective:**

This study aimed to evaluate the effect of a fall impact mitigation robot on psychological reassurance and task performance during dynamic balance tasks in healthy young adults, with a focus on self-efficacy and functional reach capacity.

**Methods:**

Twenty-four healthy adults (age: mean 28.9, SD 7.9 years) were randomly assigned to experimental (n=12) or control (n=12) groups. All participants performed a baseline functional reach test, followed by a series of progressively challenging reach tasks (starting at 98% of maximum reach and increasing by 2% until failure). The experimental group performed tasks while wearing the fall impact mitigation robot; the control group performed without it. Self-efficacy ratings (–5 to +5 scale) were recorded before each trial. Center of pressure (COP) data were continuously collected. Statistical analyses included Mann-Whitney *U* tests for self-efficacy, Kaplan-Meier survival analysis for task performance, and *t* tests for functional reach test and COP changes.

**Results:**

During reach trials ≥102% of baseline, the experimental group (median 1.0, IQR 0.0 to 3.0) reported significantly higher self-efficacy ratings than did the control group (median 0.0, IQR –1.0 to 2.0; *U*=1292.5; *P*=.047). However, no significant differences were observed in changes in functional reach capacity (experimental: mean 104.2%, SD 3.8%; control: mean 103.6%, SD 2.5%; *P*=.62) or COP displacement (experimental: mean 108.9%, SD 10.4%; control: mean 114.1%, SD 9.8%; *P*=.23). Survival analysis revealed a nonsignificant trend toward greater task persistence in the experimental group (*χ*²_1_=0.36, *P*=.55).

**Conclusions:**

The fall impact mitigation robot significantly improved self-efficacy during challenging balance tasks, despite providing no active physical support. These findings underscore the role of psychological reassurance in modulating balance-related behavior and suggest that robotic safety systems may influence motor performance through psychological mechanisms. Integrating psychological support into robotic fall prevention strategies may enhance their effectiveness.

**Trial Registration:**

UMIN Clinical Trials Registry UMIN000049284; https://center6.umin.ac.jp/cgi-open-bin/ctr_e/ctr_view.cgi?recptno=R000056126

## Introduction

The rapid aging of the global population presents major challenges to health care and social security systems. Among these, age-related declines in physical function, particularly mobility limitations, adversely affect the quality of life and independence of older adults [1]. To address these challenges, assistive robotic technologies are being developed for various domains, including mobility, transfer, bathing, and communication [2-6]. These systems are anticipated to play a key role in promoting healthy longevity by supporting physical, social, and psychological well-being [7,8].

Fall prevention is a central concern in the context of age-related functional decline, as falls compromise both safety and independence. Approximately one-third of older adults experience at least one fall annually [9,10], and the risk of fall-related injuries increases due to age-associated impairments in musculoskeletal, cardiovascular, visual, vestibular, and proprioceptive functions [11-15]. Beyond physical injury, the fear of falling contributes to a cycle of reduced mobility, decreased activity, and social withdrawal [16,17]. This fear can negatively influence balance control and movement behavior [18], particularly in older adults with physical disabilities, thereby limiting social participation and increasing the risk of future falls [19-21].

To mitigate these risks, various robotic solutions for fall prevention have been proposed. These include “smart robotic walkers” that anticipate movement patterns to provide proactive support [22] and “robotic canes” that adapt to users’ gait characteristics [23-25]. While promising, these systems are often optimized for clinical settings and may be impractical for home use due to space and mobility constraints [26]. Moreover, their potential psychological benefits remain poorly characterized, despite the crucial role of fall-related self-efficacy in fall prevention outcomes.

Recent studies have emphasized the importance of psychological factors in fall prevention. Self-efficacy, as defined by Bandura [27], refers to an individual’s belief in their ability to carry out actions necessary to achieve specific outcomes. Tinetti et al [28] introduced the concept of falls efficacy as a domain-specific form of self-efficacy, meaning the confidence to perform daily activities without falling. Building on this, Powell and Myers [29] developed the Activities-specific Balance Confidence Scale, which assesses confidence in maintaining balance during a range of activities. More recently, Soh et al [30-32] proposed a multidimensional framework for fall efficacy, encompassing 4 distinct domains across the fall continuum: balance confidence (pre-fall), balance recovery confidence (near-fall), safe-landing confidence (fall-landing), and post-fall recovery confidence (post-fall). This expanded model highlights the need for psychological resilience not only to prevent falls but also to manage their occurrence. Clinical findings suggest that such psychological constructs can directly modulate balance control mechanisms, potentially masking or compensating for physical impairments [18]. As such, effective fall prevention strategies should integrate both physical safeguards and psychological reinforcement.

Motor learning theory provides a framework for understanding how to effectively provide such psychological reinforcement. Self-efficacy influences task engagement, and learning is optimized when difficulty appropriately matches ability [27,33]. However, people naturally tend to be conservative, using only a portion of their available stability capacity due to inherent safety mechanisms [34]. This suggests that effective balance training must address both the physical and psychological barriers that limit engagement with appropriately challenging tasks.

The present study focuses on a ceiling-mounted fall impact mitigation robot designed to address both physical and psychological aspects of fall prevention. This suspended system provides continuous support during daily activities in home environments, with almost no limitations on the range of movement. While the system was primarily designed to reduce the physical risk of fall-related injuries, its impact on psychological factors, particularly fall-related self-efficacy, has not been fully elucidated. Specifically, it remains unclear whether the constant presence of such a safety system influences users’ psychological state, and if so, whether this influence translates into measurable changes in motor performance when facing such challenging tasks.

This study aims to examine the effect of the fall impact mitigation robot on psychological reassurance and task performance during dynamic balance challenges. Young healthy adults were selected as participants to establish a baseline understanding of these mechanisms, unconfounded by age-related impairments. We used the functional reach test (FRT), a validated tool for assessing dynamic balance and fall risk across age groups [35]. The FRT was particularly suited to this study, as it provides a quantitative measure of physical reaching ability and reflects the confidence required to perform challenging balance tasks [34]. Participants were asked to perform progressively demanding reaching tasks that exceeded their maximum capacity, either with or without robotic safety support depending on their group assignment. Prior to each attempt, participants were asked the self-efficacy ratings to quantify how perceived safety influences both confidence and motor performance. By investigating this relationship, we aim to elucidate how robotic safety systems modulate human movement behavior, thereby informing the development of comprehensive fall prevention strategies that promote safe, independent living among older adults.

## Methods

### Participants

Twenty-four healthy adults (12 men and 12 women; age: mean 28.9, SD 7.9 years; height: mean 1.64, SD 0.09 m; weight: mean 59.1, SD 12.5 kg) participated in this study. Participants were randomly assigned to either the experimental group (n=12; 7 men, 5 women; age: mean 27.5, SD 7.9 years; height: mean 1.65, SD 0.08 m; weight: mean 59.9, SD 13.8 kg) or the control group (n=12; 5 men, 7 women; age: mean 30.3, SD 8.1 years; height: mean 1.62, SD 0.10 m; weight: mean 58.3, SD 11.6 kg). There was no significant association between grouping and gender distribution (*χ*²_1_=0.67; *P*=.41). Statistical analysis showed that the randomization achieved an adequate gender ratio, and this slight demographic variation does not represent a methodological flaw. All participants provided written informed consent prior to the study.

### Ethical Considerations

The study protocol was approved by the Ethics and Conflict of Interest Committee of the National Center for Geriatrics and Gerontology (approval number 1636).

### Apparatus and Protocol

#### Measurement Equipment

FRT distance was measured using a dedicated device (T-2795, TOEI LIGHT Co., Ltd.). Center of pressure (COP) data were acquired using 2 force plates (BP400-600, Advanced Mechanical Technology, Inc.) operating at a sampling frequency of 1000 Hz. Data acquisition was managed via A-Cap, an AD conversion system and its accompanying software (version 1.26.1, 4Assist, Inc.).

#### Fall Impact Mitigation Robot

**Figure 1:**
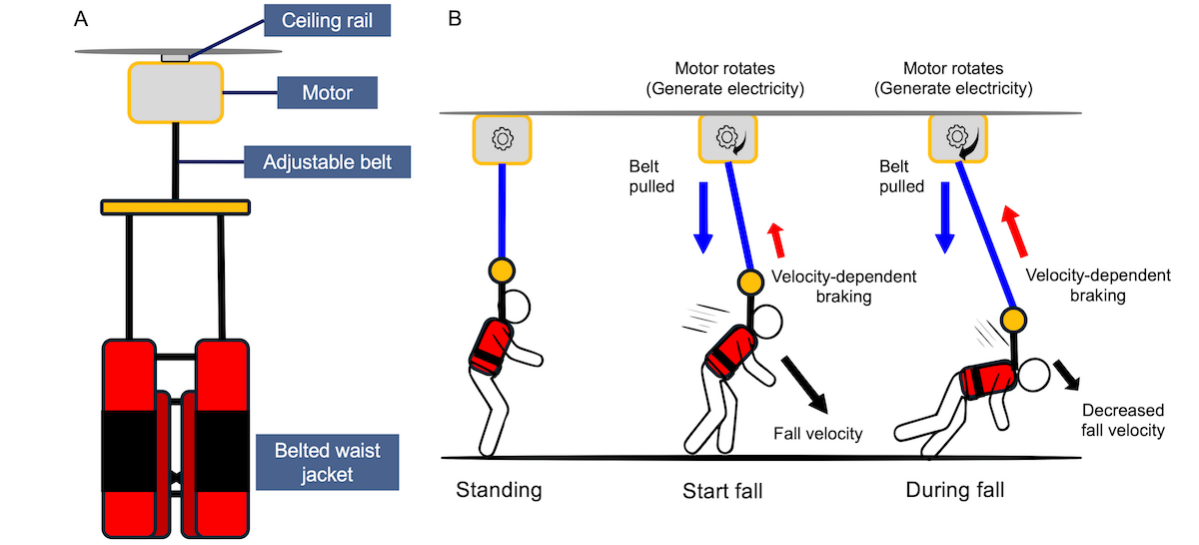
Fall impact mitigation robot system. (A) System configuration showing ceiling rail, motor unit, adjustable belt, and belted waist jacket. (B) Fall response mechanism: when a fall occurs, the adjustable belt around the motor shaft is pulled, causing motor rotation that generates electricity for velocity-dependent control to reduce fall impact during the descent sequence.

The experimental group used a ceiling-mounted fall impact mitigation robot (Yorisoi Robot, Sanyo Homes Co., Ltd.) equipped with a high-performance DC motor (model SS40E8; Figure 1). The system is permanently installed in the Living Lab, a real-world simulation environment designed for evaluating daily activities [36]. The robot remains inactive during normal movement and activates only upon detecting accelerations consistent with falling, engaging a braking mechanism to mitigate impact without interfering with normal movement.

#### Experimental Procedure

**Figure 2:**
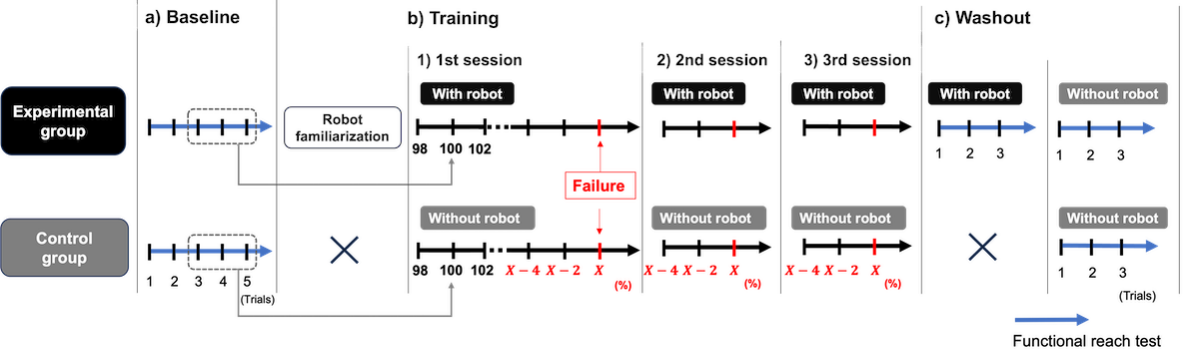
Schematic overview of the experimental procedure comparing reaching tasks performed with and without the fall impact mitigation robot.

The intervention consisted of three sequential phases (Figure 2): (1) a baseline phase to determine individual maximum reach capacity, (2) a training phase for robot familiarization and dynamic reaching tasks, and (3) a washout phase to assess residual effects without robotic assistance. To ensure participant safety throughout the experiment, a research assistant was positioned in close proximity to provide manual support if necessary, and shock-absorbing mats were placed on the floor in the forward reaching direction as a further precaution.

**Figure 3:**
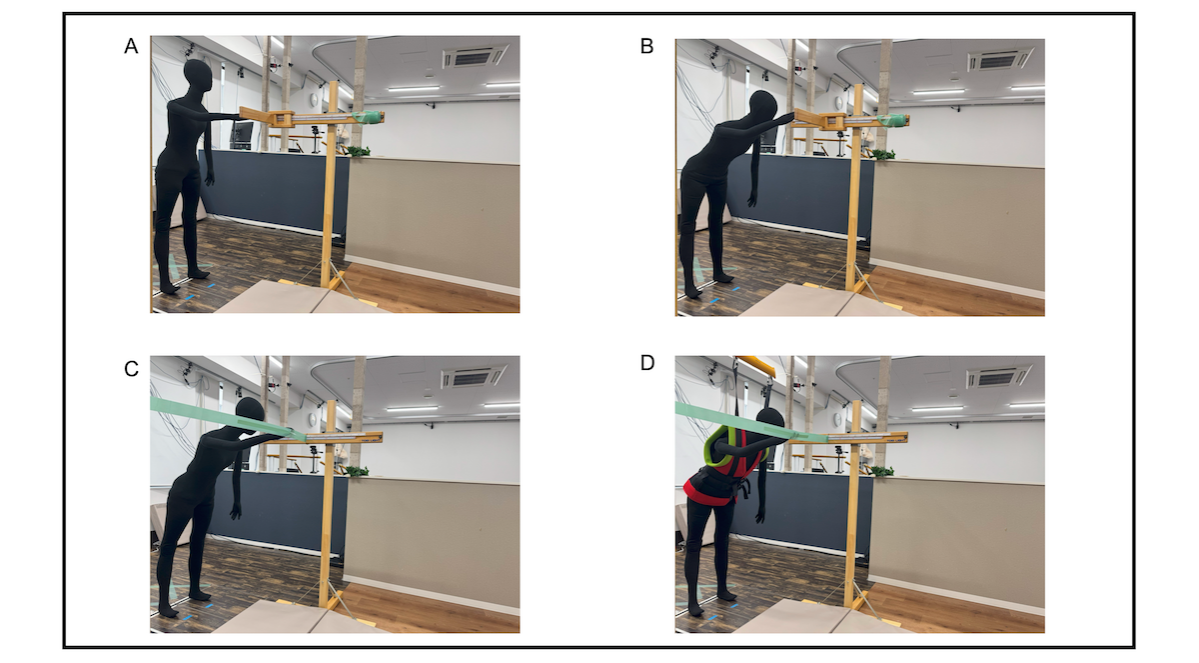
Experimental setup for the functional reach and challenging reaching tasks. (A) Initial posture for the functional reach test, with the participant standing on force plates and the measuring device aligned to acromion height. (B) Standard reach assessment, with the participant extending the right arm horizontally. (C) Challenging functional reach test, in which the participant attempts to reach beyond their baseline maximum distance. (D) Challenging reach task with the participant wearing the fall impact mitigation robot.

Baseline phase: All participants first completed a standard FRT to establish baseline performance (Figure 3A). Participants stood barefoot with feet shoulder-width apart on the force plates (right foot on right plate, left foot on left plate). The measuring device was adjusted to acromion height. From this standardized position, participants extended the right arm horizontally and pushed the measuring device forward using the extended fingers (Figure 3B). A trial was considered successful if the participant returned to the starting position without foot displacement; heel lift was permitted. After 5 trials, the average distance from the third through fifth attempts was recorded as the maximum reach distance (defined as 100%).

Training phase: The control group performed the training tasks without the robot (Figure 3C), while the experimental group performed with robotic support (Figure 3D). Participants in the experimental group were introduced to the robot’s functionality, with emphasis on its passive operation during normal movement and its role in fall impact mitigation. A researcher conducted a forward fall demonstration while wearing the robot to illustrate activation of the braking mechanism. Participants then wore the device and performed 3 controlled forward falls with robot safety support to familiarize themselves with the system’s protective function. The control group did not participate in this familiarization procedure.

Subsequently, all participants performed a series of challenging reach tasks designed to exceed the maximum reach capacity established during baseline. The same posture and setup were maintained. The task began at 98% of the individual’s maximum reach distance, increasing by 2% with each successful attempt until failure. Task failure was defined as inability to reach the target distance, any foot movement, or loss of balance preventing return to the starting position. Targets were marked with tape and reached using the extended right arm.

Before each trial, participants rated their confidence in their ability to perform the forward reaching task to the specified target distance without losing balance or moving their feet, using an 11-point scale from –5 (completely unconfident) to +5 (completely confident), with negative values indicating low confidence and positive values indicating high confidence. Upon failure, participants completed 2 additional sets of 3 trials at the failure distance and 2 easier levels (–2% and –4%). One-minute rest intervals were provided between sets to minimize fatigue.

Washout phase: After the challenging reach task, the experimental group completed 3 standard FRT trials (shown in Figures 3A and 3B) while still wearing the robot. Subsequently, both groups performed another 3 standard FRT trials without the robot to assess any residual effects (washout) of robotic support.

### Data Analysis

#### Statistical Analysis

Outliers in the FRT and COP datasets were identified and excluded using the Smirnov-Grubbs test. Data distribution was assessed using the Kolmogorov-Smirnov test for normality, followed by the Bartlett test to evaluate the homogeneity of variance across groups. Based on these preliminary assessments, parametric tests (independent samples *t* tests) were applied to normally distributed data with equal variances. When these assumptions were not met, nonparametric alternatives were used as appropriate. Statistical analyses for FRT variables were conducted using EZR software (version 1.55; Saitama Medical Center, Jichi Medical University) [37]. COP data were preprocessed in MATLAB 2022b (MathWorks) prior to statistical analysis in EZR. Analyses of the challenging reach task and self-efficacy ratings were performed using Python 3.12. A significance level of *P*<.05 was adopted for all statistical comparisons.

#### Self-Efficacy Analysis

To evaluate psychological responses, self-efficacy ratings were analyzed for all trials conducted at or beyond 102% of each participant’s baseline maximum reach. Given the ordinal nature of the self-efficacy scale (–5 to +5) as described above, between-group comparisons were performed using the Mann-Whitney *U* test.

#### Functional Reach Test Analysis

To assess the effects of the intervention on functional reach capacity, percent changes in reach distance from baseline to washout were calculated for each participant. Between-group differences in these percent changes were analyzed using independent samples *t* tests.

#### Challenging Reach Task Analysis

Performance during the challenging reach task was analyzed using Kaplan-Meier survival analysis. Reaching distances, expressed as percentages of baseline maximum, were treated as the time variable. Task failure, defined as an inability to reach the target distance, foot movement, or loss of balance, was designated as the event of interest. Survival curves for the experimental and control groups were compared using the log-rank test to determine differences in performance persistence.

#### COP Analysis

Composite COP coordinates were calculated from ground reaction force components recorded by 2 force plates. COP data were filtered using a fourth-order Butterworth low-pass filter with a 6.0 Hz cutoff frequency. The analysis window for each FRT attempt was defined by synchronizing COP trajectories with corresponding video recordings. Anteroposterior COP displacement was computed as the difference between minimum and maximum positions during baseline and washout measurements. Changes in COP displacement from baseline to washout were expressed as percentages of baseline values. Between-group comparisons of these percent changes were conducted using independent samples *t* tests.

## Results

### Self-Efficacy Analysis

**Figure 4:**
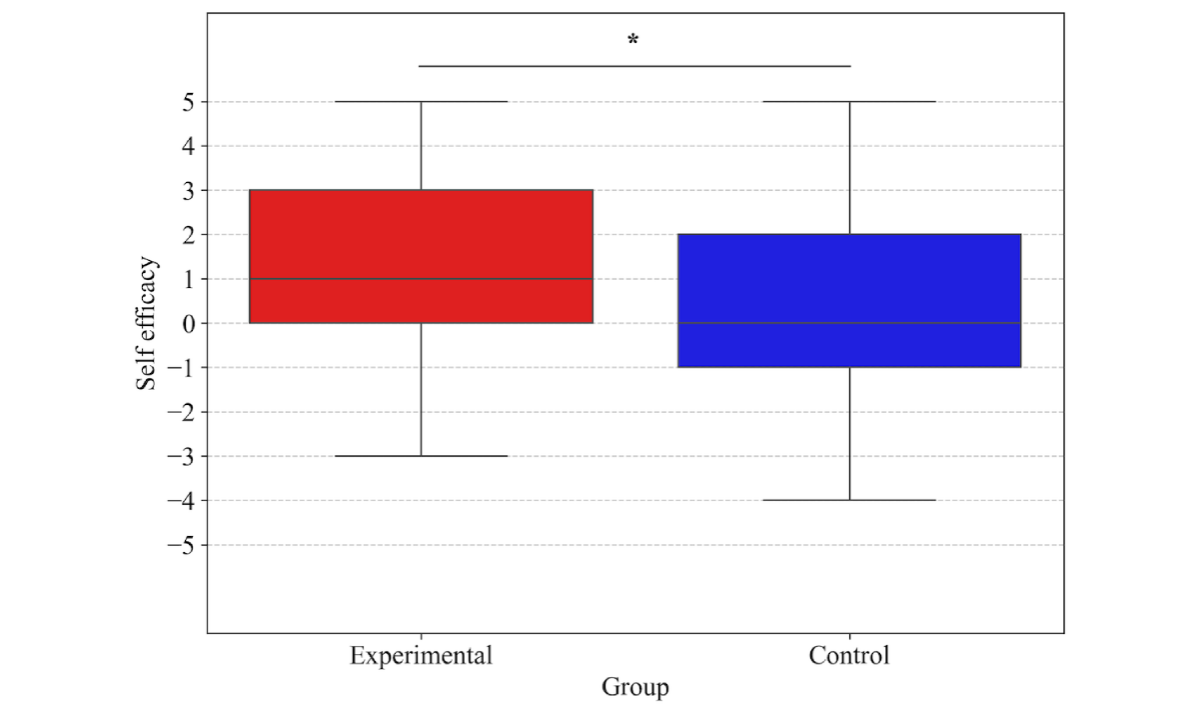
Box-and-whisker plots of self-efficacy ratings during challenging reaching tasks (≥102% of baseline maximum reach) in the experimental (red) and control (blue) groups. The y-axis represents self-efficacy scores on an 11-point scale from –5 (completely unconfident) to +5 (completely confident).

**Figure 5:**
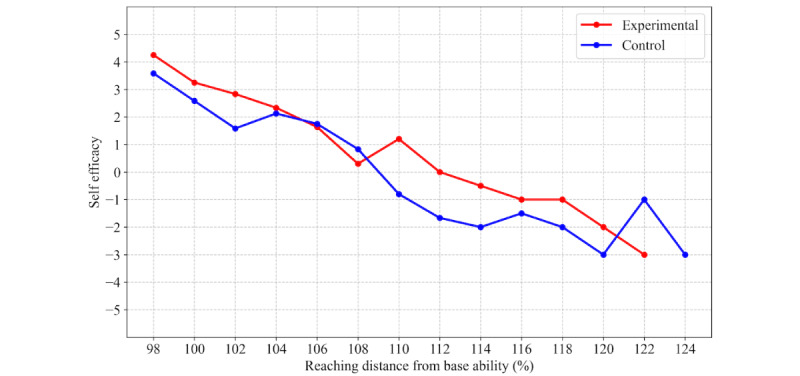
Self-efficacy ratings across different reaching distances during the challenging task for the experimental (red) and control (blue) groups. The x-axis shows reaching distance as a percentage of baseline maximum reach (100% = participant’s baseline maximum). The y-axis displays mean self-efficacy scores on an 11-point scale from –5 to +5.

Self-efficacy ratings during challenging reach trials (≥102% of baseline maximum reach) were significantly higher in the experimental group (median 1.0, IQR 0.0 to 3.0, range: –3.0 to 5.0; trials: 62) than in the control group (median 0.0, IQR –1.0 to 2.0, range: –4.0 to 5.0; trials: 53) (Figure 4). This between-group difference in Figure 4 was statistically significant (Mann-Whitney *U* test: *U*=1292.5, *P*=.047). The mean self-efficacy ratings from all participants are shown in Figure 5. Self-efficacy was higher in the experimental group (ie, when using the robot), mainly in reaching trials of 110% or more.

### The standard FRT

There were no significant between-group differences in percent changes in functional reach capacity from baseline to washout (experimental: mean 104.2%, SD 3.8%; control: mean 103.6%, SD 2.5%; *t*_22_ = 0.510, *P*=.62; Table 1). Both groups exhibited modest improvements in maximum reach distance following the intervention protocol.

**Table 1:** Comparison of physical performance measures between experimental and control groups. Data are presented as mean ± standard deviation and reflect percentage changes from baseline to washout for the functional reach test (FRT) distance and center of pressure (COP) displacement.

Outcome Measure	Experimental (n=12), mean (SD)	Control (n=12), mean (SD)	*t* value	*P* value
FRT (%)	104.2 (3.8)	103.6 (2.5)	0.510	.62
COP (%)	108.9 (10.4)	114.1 (9.8)	1.244	.23

### Challenging Reach Task

**Figure 6:**
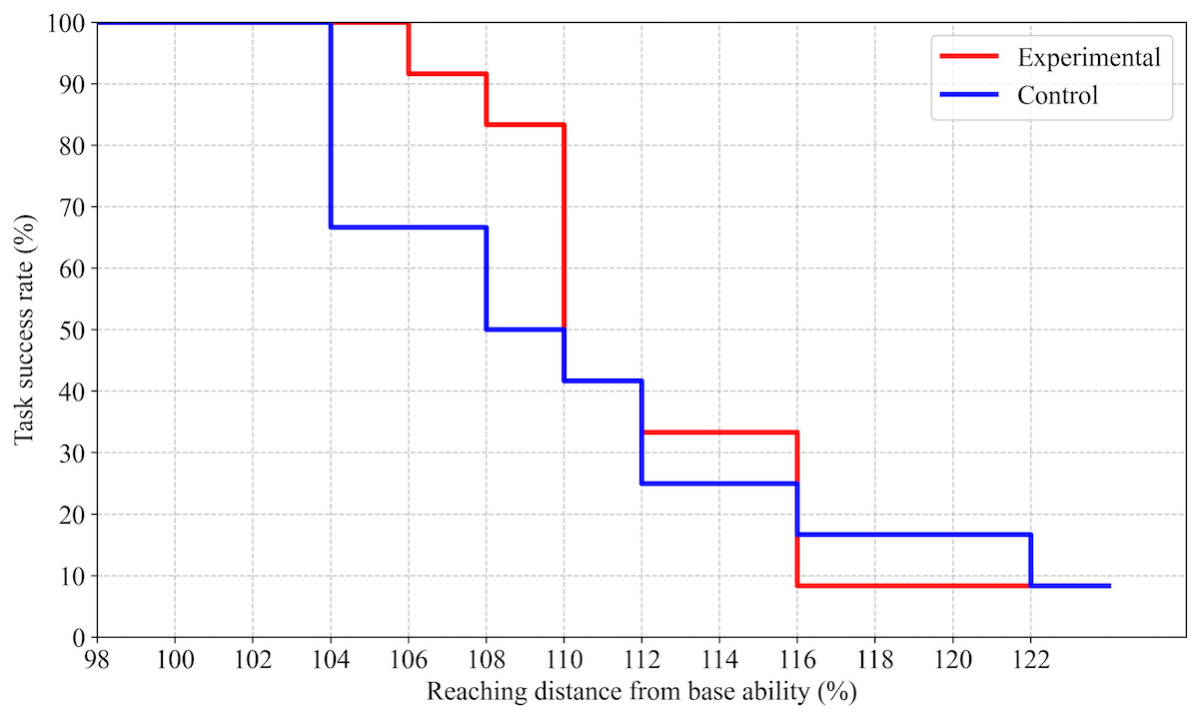
Kaplan-Meier survival curves comparing maximum reaching distances achieved during the challenging task in the experimental (red line, with fall impact mitigation robot) and control (blue line) groups. The x-axis denotes reaching distance as a percentage of baseline maximum reach. The y-axis indicates the proportion of participants able to successfully complete each reach distance.

Kaplan-Meier survival analysis of maximum reaching distances revealed no significant difference between groups (log-rank test: *χ*²_1_=0.36, *P*=.55), although the survival curve suggested a trend toward greater task persistence in the experimental group (Figure 6).

### COP Analysis

No significant differences were observed between the groups in anteroposterior COP displacement from baseline to washout (experimental: mean 108.9%, SD 10.4%; control: mean 114.1%, SD 9.8%; *t*_22_=1.244, *P*=.23; Table 1), indicating that COP dynamics during the functional reach task were not differentially influenced by the intervention.

## Discussion

### Principal Findings

This study investigated the psychological effects of a fall impact mitigation robot, focusing on its influence on self-efficacy and physical performance during balance-challenging tasks. Participants in the experimental group who wore the robot exhibited significantly higher self-efficacy ratings, particularly prior to reaching tasks that exceeded their individualized maximum reach thresholds (Figures 4 and 5). Although survival analysis suggested a trend toward greater task persistence in the experimental group (Figure 6), no statistically significant differences were observed in physical performance metrics, such as FRT scores or COP displacement (Table 1). These findings indicate that the fall impact mitigation robot provided psychological reassurance and enhanced self-efficacy, even in the absence of active robotic assistance.

### Implications for Fall Prevention

The observed enhancement in self-efficacy has direct implications for fall prevention strategies. Fear of falling contributes to activity restriction and social withdrawal, which in turn negatively impact balance control and motor behavior [18]. Among older adults, fear of falling is associated with reduced social participation and elevated risk of subsequent falls [19]. Robotic interventions that bolster self-efficacy may help interrupt this deleterious cycle. Existing evidence links higher self-efficacy with increased motivation for activities of daily living and outdoor mobility [16], suggesting that such interventions could support long-term improvements in physical activity and social engagement. However, further longitudinal research is needed to substantiate these potential benefits.

### Integration With the Falls Self-Efficacy Framework

To contextualize our findings within current theoretical models, we refer to the framework proposed by Soh et al [32], which delineates falls self-efficacy into 4 domains: balance confidence (pre-fall), balance recovery confidence (near-fall), safe-landing confidence (fall-landing), and post-fall recovery confidence (post-fall). While the fall impact mitigation robot was designed to enhance safe-landing confidence, the observed increase in self-efficacy suggests a broader psychological effect, potentially extending toward balance confidence. This cross-domain influence implies that perceived protection during potential falls may generalize to increased confidence during pre-fall activities, offering a novel direction for fall prevention strategies.

### Dissociation Between Psychological and Physical Outcomes

A key observation in this study was the dissociation between psychological and physical outcomes, namely, significant improvements in self-efficacy without parallel changes in FRT or COP displacement. As suggested by Bandura [27], enhancements in self-efficacy typically precede measurable behavioral changes, which may emerge over longer timeframes. The use of healthy young adults likely introduced ceiling effects, limiting the detection of performance-related differences. Moreover, the single-session intervention design may have been insufficient to elicit observable physical adaptations. Longitudinal studies incorporating diverse populations are needed to clarify the temporal relationship between self-efficacy and physical performance.

### Insights Into Motor Learning and Human-Robot Interaction

Our findings provide a new perspective on the application of the challenge point framework [33] to balance training. The framework posits that learning is most effective at the edge of a person’s ability. Modulating the level of physical assistance is a common approach to guide learners to this optimal state. Our results, however, demonstrate that psychological safety is a powerful, alternative modulator. The increase in self-efficacy without a change in physical performance indicates that the robot enabled participants to perform closer to their true physical limits, not by making the task easier, but by removing the fear of falling. This suggests a potential model for effective training, where psychological barriers are addressed first to allow a learner to safely engage with their optimal challenge point.

Furthermore, the enhancement of self-efficacy in the mere presence of the robot contributes to the growing body of literature on human-robot interaction. Broadbent et al [7] found that robots can fulfill both physical and psychological roles. Our results support this dual-function hypothesis, demonstrating that robotic presence alone can provide psychological reassurance and influence behavior.

### Limitations and Future Directions

Several limitations of this study warrant consideration. First, the small sample size limited statistical power and generalizability. Second, the focus on healthy young adults constrains the applicability of findings to clinical or older populations. Third, only short-term effects were assessed, leaving the long-term psychological and behavioral impacts unexamined. Fourth, the interaction between fear of falling and physical function may differ across age groups, underscoring the need for population-specific studies. Future work should incorporate validated scales aligned with the multifactorial model of falls self-efficacy proposed by Soh et al [32]. Finally, our analysis of motor behavior was restricted to coarse indicators such as COP displacement. Future investigations may benefit from high-resolution, markerless motion capture technologies, already implemented in our Living Lab environment [38], to enable fine-grained analysis of postural and movement strategies associated with robotic safety systems.

### Conclusions

These findings demonstrate that a fall impact mitigation robot can enhance self-efficacy via psychological reassurance, even in the absence of active physical assistance. This indicates that psychological support enhances self-efficacy, but that physical assistance is also necessary, highlighting the need for fall prevention strategies that integrate both. The observed enhancement in self-efficacy may promote greater engagement in physical activity and, in turn, contribute to improved functional capacity, a hypothesis that warrants validation through longitudinal investigation.

## Abbreviations

COP: center of pressure
FRT: functional reach test
